# Occupational Disruptions during the COVID-19 Pandemic and Their Association with Healthcare Workers’ Mental Health

**DOI:** 10.3390/ijerph18179263

**Published:** 2021-09-02

**Authors:** Natasha Smallwood, Amy Pascoe, Leila Karimi, Marie Bismark, Karen Willis

**Affiliations:** 1 Department of Respiratory Medicine, The Alfred Hospital, 55 Commercial Road, Prahran, VIC 3004, Australia; 2 Department of Allergy, Immunology and Respiratory Medicine, Central Clinical School, The Alfred Hospital, Monash University, Melbourne, VIC 3004, Australia; amy.pascoe@svha.org.au; 3 School of Psychology and Public Health, La Trobe University, Melbourne, VIC 3083, Australia; l.karimi@latrobe.edu.au; 4 School of Medicine and Healthcare Management, Caucasus University, Tbilisi 0102, Georgia; 5 Department of Psychiatry, Royal Melbourne Hospital, Grattan St, Parkville, VIC 3050, Australia; mbismark@unimelb.edu.au; 6 Centre for Health Policy, Melbourne School of Population and Global Health, University of Melbourne, Parkville, VIC 3050, Australia; 7 College of Health and Biomedicine, Victoria University, Footscray, Melbourne, VIC 3011, Australia; karen.willis@vu.edu.au; 8 Division of Critical Care and Investigative Services, Royal Melbourne Hospital, Grattan Street, Parkville, VIC 3050, Australia

**Keywords:** COVID-19, healthcare services, mental health, leadership, communication

## Abstract

Background: The COVID-19 crisis has caused prolonged and extreme demands on healthcare services. This study investigates the types and prevalence of occupational disruptions, and associated symptoms of mental illness, among Australian frontline healthcare workers during the COVID-19 pandemic. Methods: A national cross-sectional online survey was conducted between 27 August and 23 October 2020. Frontline healthcare workers were invited to participate via dissemination from major health organisations, professional associations or colleges, universities, government contacts, and national media. Data were collected on demographics, home and work situations, and validated scales of anxiety, depression, PTSD, and burnout. Results: Complete responses were received from 7846 healthcare workers (82.4%). Most respondents were female (80.9%) and resided in the Australian state of Victoria (85.2%). Changes to working conditions were common, with 48.5% reporting altered paid or unpaid hours, and many redeployed (16.8%) or changing work roles (27.3%). Nearly a third (30.8%) had experienced a reduction in household income during the pandemic. Symptoms of mental illness were common, being present in 62.1% of participants. Many respondents felt well supported by their workplaces (68.3%) and believed that workplace communication was timely and useful (74.4%). Participants who felt well supported by their organisation had approximately half the risk of experiencing moderate to severe anxiety, depression, burnout, and PTSD. Half (50.4%) of respondents indicated a need for additional training in using personal protective equipment and/or caring for patients with COVID-19. Conclusions: Occupational disruptions during the COVID-19 pandemic occurred commonly in health organisations and were associated with worse mental health outcomes in the Australian health workforce. Feeling well supported was associated with significantly fewer adverse mental health outcomes. Crisis preparedness focusing on the provision of timely and useful communication and support is essential in current and future crises.

## 1. Introduction

Crises such as the COVID-19 pandemic impose prolonged and extreme demands on healthcare systems and healthcare workers (HCWs). The second wave of the Australian COVID-19 outbreak occurred primarily in Melbourne, Victoria, between June and October 2020 [1], resulting in strict lockdown measures and both domestic and international border closures in anticipation of surging COVID-19 cases [2]. Among the general public, reduced paid work was common during this time [3]; up to 50% of Australians experienced financial distress [4] and UK studies of the general public identified financial and social adversity during COVID-19 as predictors for poorer mental health outcomes [5,6]. 

The effect of the pandemic and associated restrictions on Australian HCWs is unclear, with little quantitative work on the influence of occupational change and organisational leadership on the mental health of HCWs during COVID-19. Internationally, hospital leaders expressed concerns about infection risks to redeployed staff, patient safety due to HCWs operating outside their scope of training, and loss of appropriate training opportunities for HCWs [7]. Concerns have also been raised regarding delayed career progression for junior doctors in Australia due to workforce changes during the pandemic [8]. Evidence from prior infectious disease outbreaks and natural disasters indicate that management and communication styles adopted by healthcare organisations can affect the mental health of HCWs and their willingness or ability to adapt to changes [9,10,11].

This paper reports a subset of findings from the Australian COVID-19 Frontline Healthcare Workers Study, an initiative led by frontline clinicians in partnership with academics to investigate the prevalence and severity of occupational, social, and financial disruptions and their impact on mental health in Australian HCWs during the COVID-19 pandemic.

## 2. Materials and Methods

### 2.1. Study Design and Sample

A nationwide, anonymous, cross-sectional online survey of self-identifying frontline HCWs in Australia was conducted between 27 August and 23 October 2020. Australian healthcare workers were recruited through multiple strategies. Information regarding the survey was emailed to CEOs and departmental directors of frontline areas (emergency medicine, critical care, respiratory medicine, general medicine, infectious diseases, palliative care, and hospital aged care) of all public hospitals throughout Victoria and to multiple hospitals around Australia. Hospital leaders were asked to share the survey information with colleagues. Thirty-six professional societies, colleges, universities, associations, and government health department staff also disseminated information about the survey across Australia. Additionally, the study was promoted through 117 newspaper advertisements, 8 television and radio news items, and 30 social media sites. Participants did not need to have cared for people with COVID-19 to take part.

### 2.2. Data Collection

Data were collected at a single time point. Participants completed the online survey via direct link or through a purpose-built website (https://COVID-19-frontline.com.au/). Data were collected and managed using REDcap electronic data capture tools [12]. Information collected included demographics, home life details, professional background, work arrangements, the impact of the pandemic on employment and finances, organisational leadership, occupational change including redeployment (change in work area or location) and role change, exposure to COVID-19, and health and recreational habits (Supplement S1). Most questions were in single- or multiple-choice format, with free-text responses for more detailed answers. Five validated psychological measurement tools were used to assess symptoms of mental illnesses, including: anxiety (Generalised Anxiety Disorder (GAD-7)), depression (Patient Health Questionnaire (PHQ-9)), post-traumatic stress disorder (PTSD) (abbreviated Impact of Event Scale (IES-6)), and burnout (abbreviated Maslach Burnout Inventory (MBI); subdomains of emotional exhaustion, depersonalisation, and personal achievement). Burnout on the MBI is indicated by higher scores on the emotional exhaustion and depersonalisation subdomain and lower scores on the scale of personal achievement. Resilience was assessed using the abbreviated 2-item CD-RISC-2 scale. For each mental health scale, outcomes were merged into dichotomous categories as follows: depersonalisation: 0–3 low, 4–18 moderate to high; emotional exhaustion: 0–6 low, 7–18 moderate to high; personal achievement: 0–13 low; 13–18 moderate to high [13]; IES: 0–9 minimal to none, >9 moderate to severe [14]; GAD7: 0–9 none to mild, 10–21 moderate to severe [15]; PHQ9: 0–9 none to mild, 10–27 moderate to severe [16]. Ethics approval was provided by the Royal Melbourne Hospital Human Research Ethics Committee (HREC/67074/MH-2020, approved 20th August 2020).

### 2.3. Statistical Analysis

A power calculation for multiple linear models was computed using RStudio [17]. With an expected medium to large effect size, a power of 0.95, and significance level of 0.05, 6348 participants were required. Data analysis was performed using SPSS statistical software version 26.0 (IBM Corp, Armonk, NY, USA). Demographic and socioeconomic characteristics are reported descriptively. Individual outcomes examined included: increased paid hours, increased unpaid hours, redeployment, role change, and mental health symptoms (measured on validated scales). Predictors of occupational disruption or worse mental health outcomes were identified through univariate logistic regression models then entered into multivariate logistic regression models. Associations are presented as odds ratios (ORs) with 95% confidence intervals (CIs).

Predictor variables examined in regression models for occupational disruptions and training/confidence included: age, sex, state, occupation, number of years working since graduating, lives alone, lives with children, lives with people aged over 65, frontline area, practice location, works with COVID-19 patients, anticipates working with COVID-19 patients, received PPE training, need for additional training, confidence in training, close friends or relatives infected with COVID-19, changed household income, concerns regarding household income, and pre-existing mental health condition. Predictor variables examined in regression models for mental health outcomes were current employment, change in employment, change in paid or unpaid work hours, redeployment, change in job role, training to care for patients with COVID-19, confidence to care for people with COVID-19, confidence in PPE usage, need for additional training, satisfaction with workplace communication, and satisfaction with organisational support. Reference categories for predictor variables are stated in results tables. *p* < 0.05 was taken to indicate statistical significance.

## 3. Results

A total of 9518 responses were returned, with complete responses from 7846 participants reported. Most participants resided in the Australian state of Victoria (6685, 85.2%). Reflecting the demographics of the Australian workforce (75% female) [18,19,20], most participants were women (6344, 80.9%) (Table 1). Nearly half the participants (2725, 41.1%) had more than 15 years of work experience since graduation. Most participants (6759, 86.2%) lived with at least one other person; a third (2744, 34.9%) had children at home, and 8.9% (697) had someone aged over 65 years (“elders”) living with them.

### 3.1. Occupational Environment and Disruptions

Over a third (3063, 39.0%) of participants were currently working with people infected with COVID-19, and two-thirds (2891, 60.5%) anticipated working with people infected with COVID-19 in the future. Nearly half (42.3%) of participants reported working increased paid (20.8%) or unpaid (21.5%) hours in their role. Redeployment (16.8%) or a change in work role (27.3%) were not uncommon (Table 2). Three-quarters (5883, 74.4%) believed they had received timely and useful communication regarding the pandemic from their organisations, and two thirds (5352, 68.3%) believed their workplace actively supported their well-being and mental health during the pandemic.

### 3.2. Predictors of Occupational Change

In the multivariate regression model, independent predictors of experiencing a change to work role included: being a nurse or allied health worker, working in other frontline areas (including paramedicine, radiology, pharmacy, pathology and clinical laboratories, or other areas compared to ED), and having a prior mental health diagnosis (Table 3). Independent predictors of being redeployed during the pandemic included having less than 5 years’ work experience, having a friend or family member infected with COVID-19, living with three or more people aged over 65, having concerns about income, being in a nursing or allied health role, currently working with COVID-19 patients, and certain frontline areas (anaesthetics, perioperative or surgical, medical specialties, other non-medical roles, and primary care). People with 6–10, 11–15, and ≥15 years’ experience since graduation (vs. ≤5 years), with children at home, and reporting altered income, had reduced odds of being redeployed.

Independent predictors of reporting working additional paid hours since the pandemic commenced included: female sex, age between 20–30, 31–40, and 41–50 (compared to over 50 years), and in a nursing role. By contrast, people working in allied health and certain frontline areas (anaesthetics, perioperative or surgical, and medical specialty areas compared to ED staff) had reduced odds of working increased paid hours. 

Independent predictors of reporting working additional unpaid hours included having a family member or friend infected with COVID-19, and working in certain frontline areas (primary care, a medical speciality, or a non-medical frontline area). The odds of working additional unpaid hours increased with years of experience since graduation. Nurses and allied health workers (compared to doctors) had reduced odds of working additional unpaid hours.

### 3.3. Predictors of Individual Preparedness

Independent predictors for receiving training to care for people with COVID-19 included: nursing role, working in ICU, and currently working with people infected with COVID-19. Women, allied health staff and people working in certain frontline areas (primary care and other frontline areas) had reduced odds of receiving training (Table 4).

Independent predictors for having confidence in their ability to care for people with COVID-19 included: living in the Australian state of Victoria, having 11–15 or more than 15 years’ experience since graduating, nursing role, working in ICU, and currently working with COVID-19 patients. Women, those age 41–50 years (compared with over 50), people with a prior mental health diagnosis, people working in a regional area (compared to metropolitan), and certain frontline areas (anaesthetics, perioperative or surgical, other non-medical roles, and primary care) had reduced odds of having confidence in caring for people with COVID-19.

Independent predictors of being confident using PPE included: living in the Australian state of Victoria, nursing role, and currently working with people infected with COVID-19. Conversely, people aged 30–50 years (compared with over 50), those with prior mental health diagnoses, living in regional areas, and primary care workers had reduced odds of being confident using PPE. Independent predictors of desiring further training regarding COVID-19 management or PPE usage included: female sex, working in regional locations, and working in primary care. People in age categories under 50 years, living in Victoria, working in ICU, with nursing, allied health or other roles (compared to doctors), and those currently working with COVID-19 patients had reduced odds of desiring further training.

Independent predictors of being less confident in a new area after redeployment included: having a friend or family member infected with COVID-19, nursing role, and working in anaesthetics, perioperative, or surgical areas. There was no association between other personal or occupational co-variates and confidence after redeployment.

Independent predictors of being confident in a new role after role change related to the pandemic included: having 6–10 years of postgraduate experience or over 15 years postgraduate experience. People working in anaesthetics, perioperative, or surgical areas had reduced odds of being confident in a new role. There was no association between other personal or occupational co-variates and confidence after role change.

### 3.4. Occupational Change and Mental Health

A third of participants (2389, 30.4%) had a diagnosed mental health condition prior to the pandemic and many experienced self-reported mental health concerns since the pandemic, primarily anxiety (4875, 62.1%), burnout (4575, 58.3%), and depression (2175, 27.7%). Validated scale-assessed symptoms of moderate to severe anxiety or depression were seen in a third of participants (anxiety 2216, 28.3%; depression 2192, 28.0%). Burnout symptoms were particularly prevalent, with 70.9% (5458) experiencing moderate to severe emotional exhaustion and 37.4% (2877) experiencing moderate to severe depersonalisation. A third of participants scored low for the personal achievement subdomain of burnout (2243, 29.1%). Self-reported PTSD was relatively low at 5.4% (427), though nearly half (3155, 40.5%) showed moderate to severe symptoms of PTSD in the validated IES-6 instrument. 

**Figure 1:**
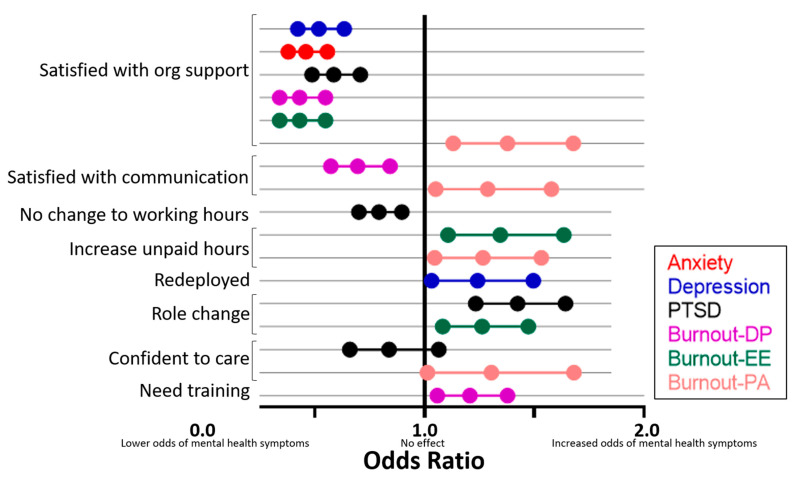
Bars are indicative of odds ratio and 95% confidence intervals. Colour key represents predictors for each validated scale outcome: Anxiety (Generalised Anxiety Disorder Scale); Depression (Patient Health Questionnaire); PTSD (post-traumatic stress disorder; Impact of Events Scale); Burnout (Maslach Burnout Inventory; DP = Depersonalisations; EE = Emotional Exhaustion; PA = Personal Accomplishment). Lower odds ratio for PA indicates poorer outcomes. Reference categories—satisfied with organisational support vs. neutral, satisfied with workplace communications vs. neutral, no change to working hours vs. change, increased unpaid hours vs. negative response, redeployed vs. negative response, role change vs. negative response, confident to care vs. neutral, needs training vs. negative response.

Perception of workplace support was the most frequent mediator of mental health outcomes (Figure 1 and Supplementary Table S1). Feeling well supported at work was significantly associated with reduced odds of experiencing moderate to severe mental health symptoms on all measured outcomes (anxiety: OR 0.46, CI 0.38–0.56, *p* = 0.001; depression: OR 0.52, CI 0.42–0.63, *p* = 0.001; PTSD: OR 0.59, CI 0.49–0.71, *p* = 0.001; depersonalisation subdomain of burnout: OR 0.59, CI 0.49–0.71, *p* = 0.001; and emotional exhaustion subdomain of burnout: OR 0.43, CI 0.34–0.55, *p* < 0.001). Feeling well supported at work was significantly associated with increased odds of experiencing personal achievement (OR 1.38, CI 1.13–1.68, *p* = 0.001). Satisfaction with the usefulness and timing of workplace communications was independently associated with reduced odds of depersonalisation (OR 0.70, CI 0.57–0.84, *p* < 0.001) and increased odds of personal achievement (OR 1.29, CI 1.05–1.58, *p* = 0.015).

Redeployment was independently associated with increased odds of depression (OR 1.24, CI 1.03–1.50, *p* = 0.022), and role change was independently associated with increased odds of PTSD (OR 1.42, CI 1.23–1.64, *p* < 0.001) and emotional exhaustion (OR 1.26, CI 1.08–1.47, *p* = 0.003). Working additional unpaid hours since the onset of the pandemic was independently associated with increased odds of experiencing emotional exhaustion (OR 1.35, CI 1.11–1.64, *p* = 0.003) and reduced odds of higher personal achievement (OR 1.27, CI 1.05–1.53, *p* = 0.015). Experiencing no change to working hours (OR 0.79, CI 0.70–0.90, *p* = 0.001) and being confident caring for COVID-19 patients (OR 0.82, CI 0.70–0.98, *p* = 0.032) reduced the odds of PTSD. Similarly, feeling confident using PPE increased the odds of personal achievement (OR 1.31, CI 1.01–1.68, *p* = 0.039). Those who indicated a need for additional training in PPE usage had greater odds of experiencing depersonalisation (OR 1.21, CI 1.06–1.38, *p* = 0.005).

## 4. Discussion

This is the largest, cross-sectional study globally to quantitatively measure the impacts of the COVID-19 pandemic on occupational disruption, pandemic preparedness, and mental health, as reported by Australian frontline HCWs. Disruption to healthcare services during the pandemic has been profound, with many workers experiencing changes in their working hours, job role, and household income. Although HCWs in direct contact with COVID-19 patients were more likely to report having received adequate training regarding COVID-19 patient care and PPE use, many workers felt a need for additional training and lacked confidence in their ability to care for patients and use PPE safely. A significant portion of participants was unsatisfied with the support or communication provided by their workplaces during this time. Personal, social, and professional predictors have been identified for occupational change, individual preparedness, and mental health problems.

### 4.1. Prevalence of Occupational Change and Individual Preparedness

Working increased unpaid hours during COVID-19 was associated with working as a doctor and additional years of experience since graduation, which is a surrogate for seniority in the role. The current survey did not identify what type of work the additional unpaid hours constituted. However, these additional hours may reflect increased pandemic response coordination and leadership responsibilities. While working unpaid hours has been the ‘norm’ for doctors for some time [21], this practice is untenable and has long-term consequences on mental health and workforce retention [22,23]. Furthermore, there may be legal ramifications, as expecting people to work unpaid is unlawful [24]. In Australia, the junior doctor workforce recently launched legal action to challenge this practice and demand payment for all hours of work [25]. 

People with children at home were not more likely to change their employment status or work hours after the onset of COVID-19. This may be reflective of policy choices in Victoria to keep schools and childcare open for frontline workers, reducing their need to undertake additional caring responsibilities [26]. A policy implication statement prepared by McHugh [27] indicates that current US emergency response plans rely on an overestimation of nursing capacity based on the presumed availability of temporary or pool nurses who often also hold a permanent appointment elsewhere. Given the higher likelihood of nurses to have increased their paid hours during COVID-19, it is likely that Australian healthcare systems rely on a similarly overstretched workforce.

Training was more likely to be available to those who currently worked with COVID-19 patients as well as HCWs in the Australian state of Victoria, which was where the majority of cases during the second wave of the Australian pandemic occurred. The need for additional training expressed by half of the participants and the high rates of people anticipating working with COVID-19 patients in the future indicates that training may be reactive rather than proactive. Insufficient pre-emptive training in preparation for crisis situations as a barrier to HCW preparedness is not unique to Australian HCWs or the COVID-19 pandemic [10,28,29,30]. Competency-based training programs for non-technical skills have been shown to significantly improve learning outcomes [31] and increase HCW confidence in resource management when faced with a crisis situation [32]. The rapid development of the COVID-19 outbreak in Australia limited the ability to pre-emptively train HCWs beyond those in the most direct contact, though lessons from this pandemic should inform competency-based programs in preparation for ongoing and future infectious disease outbreaks.

### 4.2. Impact on Mental Health

Two key occupational factors emerged in the analysis of predictors for poorer mental health outcomes during COVID-19: perception of workplace support and communication and availability of training or confidence in caring for people infected with COVID-19. Feeling well supported by their workplace was a key independent predictor of mental health outcomes, being associated with approximately half the risk of experiencing moderate to severe anxiety, depression, burnout, and PTSD. The importance of workplace support is well established in the literature [9,33,34]. Although workplace support has an important buffering effect on adverse mental health outcomes during crises for HCWs, these supports are often unavailable or under-utilised. The current study found that 16.2% (875) of participants felt somewhat or very unsupported. This is consistent with surveys showing that 12% of Belgian HCWs had a negative experience when seeking support from their workplace during COVID-19, and 15% did not seek support at all despite feeling a need to do so [34].

In addition to support, the communications provided by healthcare organisations are an opportunity to safeguard HCWs. Those who were satisfied with the usefulness and timeliness of communications were less likely to experience depersonalisation and more likely to have a higher sense of personal achievement. Unclear communications have previously been shown to erode HCWs’ sense of trust in their workplace leadership and reduce their willingness to respond in a crisis [35,36,37]. Empowering nurses to hold leadership roles during crises is essential, as they occupy a unique space, are trained in emotional support and conflict resolution, and may have insights that are not apparent to non-frontline workers [33,38].

Those who experienced changes in their work role or were redeployed to a new work area were at increased risk of depression, PTSD, and emotional exhaustion. Similarly, those working additional unpaid hours were more likely to experience emotional exhaustion. This is consistent with prior work on Australian HCW cohorts indicating that the risk of experiencing a common mental disorder doubles when work hours exceed 55 h per week [23], and each additional hour represents a 3% increase in the odds of experiencing psychosocial distress [39]. The need to redeploy or alter the work roles of some HCWs during a crisis are likely unavoidable, though the increased risk of poorer mental health outcomes for these workers highlights the need to ensure adequate supports are available. Targeted training and mental health support for these workers may help reduce the impact of crises such as COVID-19 on these workers [40].

HCWs who indicated a need for additional training were more likely to experience depersonalisation, whilst those who felt confident to care for COVID-19 patients were less likely to experience PTSD symptoms. This is consistent with studies showing that HCWs performing tasks outside their scope of training due to resource scarcity are at increased risk of distress [41]. Although training opportunities are generally well provided, there may be a gap in the communication about, or accessibility of, these training opportunities to all HCWs. Previous work has shown that despite high levels of willingness, up to a third of HCWs are unaware of where or how to access additional training [10].

### 4.3. Strengths and Limitations

The large sample size in this study enabled a detailed examination of occupational change and the association with mental health. The demographic breakdown of the participants was broadly female, though this is representative of the Australian workforce, which is 75% female [18,19,20]. The survey did not specifically enquire about increased at-home duties as a form of unpaid working hours, and future studies should address this, as women and caregivers are more likely to perform high levels of unpaid work in the home, particularly during COVID-19 [42,43].

Calculation of response rate was not possible due to the wide dissemination of the survey. Selection and response bias in this voluntary survey may result in over- or under-reporting of occupational change or mental health symptoms. Validated surveys to detect symptoms of mental illness were used in lieu of clinical diagnoses, as is standard practice in surveys exploring psychosocial effects of COVID-19 on health workers [44,45,46]. It was not possible to establish baseline data on mental health symptoms pre-pandemic in this cohort of Australian frontline workers due to the unexpected nature of the pandemic. However, in this study, the prevalence estimates of mental health conditions reported during the pandemic are much higher than those reported in earlier studies in non-pandemic times [47,48,49,50]. The single time point was selected to minimise the burden on HCWs, although this precludes longitudinal examination of mental health trends during the pandemic. Longitudinal research is urgently required to better understand any persisting occupational changes and associated psychosocial effects on HCWs. Occupational change and associated worsening of mental health may have important ramifications for patient safety and workforce retention.

## 5. Conclusions

This study reveals significant disruption to the working conditions of Australian frontline HCWs during COVID-19 with associated impacts on mental health. Useful and timely organisational support and communication were two of the strongest factors in mitigating impacts on mental health. Long-term, evidence-based policies and practices that focus on organisational and personal preparedness are needed to safeguard the healthcare workforce during the ongoing COVID-19 pandemic and in future crises 6.

## Figures and Tables

**Table 1:** Participants’ characteristics.

Characteristic	Frequency (*n* = 7846)	Percent (%)
**Age (years)**		
20–30	1860	23.7
31–40	2250	28.7
41–50	1738	22.2
>50	1998	25.5
**Gender**		
Male	1458	18.6
Female	6344	80.9
Non-binary	19	0.2
Prefer not to say	25	0.3
**State**		
Victoria	6685	85.2
All other Australian states	1161	14.8
**Location of practice**		
Metropolitan	6373	81.2
Regional	1407	17.9
Remote	66	0.8
**Occupation**		
Nursing	3088	39.4
Medical *	2436	31.1
Allied health	1314	16.7
Administrative staff	485	6.2
Other roles **	523	6.7

* Medical staff comprised 389 general practitioners, 1221 senior medical staff, 745 junior medical staff, and 81 students. ** Other: pharmacists: 185, clinical laboratory scientists or technicians: 176, paramedics: 95, support staff (including cleanings, security, facilities management personnel): 43, leadership role: 9, and other role: 15.

**Table 2:** Occupational disruptions and perception of workplace.

Characteristic	Frequency (*n* = 7846)	Percent (%)
**Employment status BEFORE pandemic**		
Full-time	3783	48.2
Part-time	3654	46.6
Casual/other	409	5.2
**CURRENT employment status**		
Full-time	3818	48.7
Part-time	3642	46.4
Casual/other	386	4.9
Change in work status (full-time, part-time, or casual) *		
Step up in work status	400	5.1
Step down in work status	354	4.5
**Health organisation type**		
Public	5605	76.8
Community	1140	15.6
Private	260	3.6
Other **	293	4.0
Any change in working hours since the pandemic commenced ***		
Increased paid work hours	1634	20.8
Increased unpaid work hours	1686	21.5
Decreased paid or unpaid work hours	886	11.3
No change	4039	51.5
**Household income altered due to COVID-19 pandemic**		
Increased	820	10.5
Decreased	2415	30.8
No change	4611	58.8
**Concerned about household income due to COVID-19 pandemic**	2416	30.8
**Redeployed to a new area of work**	1318	16.8
Confidence working in new work area ****	5.0	1.6
**Change in work role**	2139	27.3
Confidence working in new role ****	5.2	1.3
**Received training to care for patients with COVID-19**	2792	35.6
**Received training on PPE during the pandemic**	5137	65.5
**Needs more training regarding PPE or managing people with COVID-19**	3001	50.4
**Exposed to confirmed/suspected COVID patients (*n* = 7832 responses)**	4561	58.2
**Communication received from the workplace during the pandemic has been useful and timely**		
Strongly or somewhat agree	5833	74.4
Neither agree nor disagree	801	10.2
Strongly or somewhat disagree	1212	15.4
**Believed their workplace actively supported their well-being and mental health during the pandemic**		
Very well or somewhat supported	5352	68.3
Neither supported nor unsupported	1219	15.5
Very or somewhat unsupported	1275	16.2

* Refers to change from casual/other to part-time/full-time and vice versa. ** Other categories: examples of optional free-text responses included 67 university/research institutes, 58 ambulance/paramedic stations, 19 government or non-profit organisations, 9 correctional facilities, 13 pathology sites. *** Multiple response options could be selected. **** Confidence was scored on a 7- point scale, with 1 being the lowest confidence and 7 being highest confidence. Data given as mean and SD.

**Table 3:** Predictors of occupational disruption.

Characteristics	Increased Paid Hours		Increased Unpaid Hours		Redeployed		Work Role Changed	
	OR (95% CI)	*p*	OR (95% CI)	*p*	OR (95% CI)	*p*	OR (95% CI)	*p*
**Personal predictors**								
**Female gender**	1.32 (1.08–1.61)	0.006		N/A		N/A	1.20 (1.00–1.44)	0.046
**Age (years)**								
20–30	1.45 (1.07–1.98)	0.018	1.21 (0.82–1.79)	0.344	1.15 (0.86–1.54)	0.342		N/A
31–40	1.36 (1.04–1.77)	0.023	1.28 (0.95–1.72)	0.105	1.11 (0.85–1.44)	0.446		N/A
41–50	1.44 (1.16–1.78)	0.001	1.19 (0.94–1.52)	0.151	1.25 (1.00–1.57)	0.052		N/A
**State (VIC)**	0.98 (0.79–1.21)	0.838	0.83 (0.68–1.02)	0.077		N/A		N/A
**Prior mental health condition**		N/A		N/A		N/A	1.23 (1.07–1.42)	0.004
**Experienced family or friend infected with COVID-19**		N/A	1.27 (1.06–1.52)	0.009	1.19 (1.03–1.37)	0.016	1.15 (0.99–1.34)	0.065
**Number of years’ experience since graduating**								
5–10	0.89 (0.72–1.10)	0.277	1.55 (1.13–2.12)	0.007	0.69 (0.56–0.85)	<0.001		N/A
11–15	0.68 (0.52–0.90)	0.007	2.10 (1.47–2.99)	<0.001	0.72 (0.55–0.93)	0.012		N/A
≥15	0.79 (0.60–1.04)	0.089	3.02 (2.11–4.32)	<0.001	0.66 (0.51–0.86)	0.002		N/A
**Number of children**								
1–2		N/A	1.19 (0.98–1.44)	0.086	0.82 (0.69–0.98)	0.031		N/A
3+		N/A	0.91 (0.64–1.28)	0.575	0.99 (0.74–1.33)	0.950		N/A
**Elderly care**								
1–2		N/A	0.92 (0.69–1.23)	0.579	0.96 (0.74–1.24)	0.731		N/A
3+		N/A	0.54 (0.12–2.55)	0.437	2.66 (1.24–5.71)	0.012		N/A
**Household income altered**								
Increased	0.05 (0.04–0.07)	<0.001	0.83 (0.61–1.13)	0.229	0.59 (0.47–0.75)	0.001	0.53 (0.42–0.68)	0.001
Decreased	0.05 (0.04–0.06)	<0.001	0.77 (0.58–1.03)	0.079	0.56 (0.46–0.68)	0.001	0.40 (0.32–0.49)	0.001
Has concerns about income	1.02 (0.85–1.21)	0.851		N/A	1.25 (1.06–1.47)	0.009	1.13 (0.96–1.32)	0.140
**Professional predictors**								
**Occupation**								
Nursing	1.37 (1.15–1.63)	0.001	0.54 (0.44- 0.67)	<0.001	1.53 (1.29–1.80)	0.001	1.47 (1.21–1.77)	0.001
Allied health	0.71 (0.56–0.90)	0.005	0.69 (0.54–0.87)	0.002	1.652 (1.34–2.04)	0.001	1.90 (1.55–2.33)	0.010
Other roles	0.96 (0.67–1.38)	0.814	0.75 (0.53–1.08)	0.120	0.97 (0.67–1.42)	0.882	1.54 (1.24–1.93)	0.001
**Frontline area**								
ICU	0.90 (0.70–1.16)	0.420	1.44 (0.79–2.61)	0.236	0.89 (0.65–1.23)	0.497	0.79 (0.47–1.32)	0.365
Anaesthetics and surgery	0.51 (0.38- 0.66)	<0.001	1.13 (0.69–1.85)	0.621	3.52 (2.67–4.64)	0.001	1.06 (0.73–1.53)	0.776
Medical specialty areas	0.70 (0.58–0.85)	<0.001	1.83 (1.18–2.84)	0.007	3.53 (2.80–4.46)	0.001	1.03 (0.74–1.42)	0.878
Other *	0.87 (0.64–1.18)	0.375	2.89 (1.76–4.75)	0.001	3.24 (2.36–4.46)	0.001	1.33 (0.92–1.92)	0.130
Primary care, community and residential aged care	0.77 (0.59–1.00)	0.051	1.84 (1.15–2.93)	0.011	2.44 (1.81–3.29)	0.001	1.21 (0.86–1.69)	0.272
**Currently works with COVID-19 patients**		N/A		N/A	1.90 (1.64–2.21)	0.001		N/A
**Anticipates working with COVID-19 patients**		N/A	1.17 (0.98–1.40)	0.089		N/A	1.05 (0.91–1.22)	0.499

Outcome comparators: part-time (vs. full-time), increased paid hours (vs. negative response), increased unpaid hours (vs. negative response), redeployed (vs. negative response), and work role changed (vs. negative response). Reference categories: female gender (vs. male), age (vs. over 50 years), Victoria (vs. other states), has prior mental health condition (vs. none), experienced family or friend infected with COVID-19 (vs. none), number of years experience (vs. over 15), number of children (vs. 0), elderly care (vs. none), altered household income (vs. no change), occupation (vs. medical), frontline area (vs. emergency department), currently working with COVID-19 (vs. negative response), and anticipates working with COVID-19 (vs. negative response). * Other for the frontline area included people working in paramedicine, radiology, pharmacy, pathology and clinical laboratories, or other areas.

**Table 4:** Predictors of training and confidence.

Characteristics	Training to Care		Confident to Care for People with COVID-19		Confident in Using PPE		Need Training	
	OR (95% CI)	*p*	OR (95% CI)	*p*	OR (95% CI)	*p*	OR (95% CI)	*p*
**Personal Predictors**								
**Female gender**	0.66 (0.58–0.76)	<0.001	0.64 (0.51–0.80)	<0.001		N/A	1.36 (1.21–1.61)	<0.001
**Age (years)**								
20–30	1.00 (0.85–1.17)	0.996	0.80 (0.56–1.16)	0.248	0.80 (0.62–1.03)	0.082	1.64 (1.40–1.94)	<0.001
31–40	0.99 (0.84–1.15)	0.906	0.73 (0.54–1.00)	0.057	0.64 (0.51–0.81)	0.001	1.53 (1.31–1.79)	<0.001
41–50	1.05 (0.88–1.25)	0.556	0.66 (0.51–0.85)	0.001	0.60 (0.46–0.77)	0.001	1.44 (1.22–1.69)	<0.001
**State (VIC)**		N/A	1.51 (1.20–1.90)	0.001	1.96 (1.60–2.39)	0.001	0.64 (0.54–0.76)	0.001
**Has prior mental health condition**		N/A	0.78 (0.65–0.92)	0.005	0.75 (0.64–0.89)	0.001		N/A
**Experienced family or friend infected with COVID-19**	1.13 (1.01–1.27)	0.026	1.00 (0.84–1.19)	0.955	0.95 (0.80–1.12)	0.504	1.10 (0.98–1.24)	0.098
**Regional location**	0.99 (0.86–1.15)	0.951	0.78 (0.63–0.98)	0.033	0.81 (0.67–0.99)	0.035	1.25 (1.07–1.45)	0.004
**Household income altered**								
Increased		N/A	0.78 (0.56–1.08)	0.147	0.73 (0.54–0.99)	0.041	1.11 (0.91–1.36)	0.314
Decreased		N/A	0.77 (0.57–1.02)	0.077	0.86 (0.66–1.14)	0.296	1.12 (0.94–1.34)	0.220
**Has concerns about income**	0.89 (0.79–1.01)	0.075	0.64 (0.52–0.78)	0.001	0.63 (0.52–0.75)	0.001	1.56 (1.36–1.79)	0.001
**Professional predictors**								
**Number of years’ experience since graduating**								
6–10		N/A	1.22 (0.95–1.56)	0.118		N/A		N/A
11–15		N/A	1.50 (1.10–2.05)	0.010		N/A		N/A
≥ 15		N/A	1.81 (1.31–2.50)	0.001		N/A		N/A
**Occupation**								
Nursing	1.55 (1.37–1.76)	0.001	1.54 (1.26–1.88)	<0.001	1.56 (1.29–1.89)	<0.001	0.49 (0.43–0.56)	0.001
Allied health	0.60 (0.50–0.73)	0.001	1.09 (0.84–1.42)	0.508	1.18 (0.91–1.52)	0.216	0.52 (0.43–0.63)	0.001
Other roles	0.28 (0.22–0.35)	0.001	0.80 (0.53–1.22)	0.313	0.99 (0.75–1.33)	0.967	0.71 (0.57–0.88)	0.002
**Frontline area**								
ICU	2.51 (2.03–3.11)	0.001	1.44 (1.00–2.07)	0.048	1.03 (0.75–1.42)	0.851	0.62 (0.50–0.76)	<0.001
Anaesthetics and surgery	1.08 (0.88–1.32)	0.448	0.55 (0.40–0.75)	0.001	0.81 (0.60–1.10)	0.177	0.99 (0.81–1.22)	0.946
Medical specialty areas	0.86 (0.74–1.01)	0.079	0.80 (0.62–1.05)	0.111	0.99 (0.78–1.27)	0.947	1.05 (0.89–1.23)	0.573
Other *	0.81 (0.64–1.04)	0.104	0.65 (0.44–0.96)	0.034	0.96 (0.67–1.38)	0.823	0.86 (0.67–1.10)	0.237
Primary care, community and residential aged care	0.41 (0.33–0.51)	0.001	0.44 (0.31–0.62)	0.001	0.59 (0.44–0.79)	0.001	1.60 (1.29–1.98)	<0.001
**Currently works with COVID-19 patients**	3.32 (2.95–3.74)	0.001	2.76 (2.28–3.33)	0.001	1.61 (1.34–1.93)	0.001	0.52 (0.46–0.59)	0.001

Outcome comparators: training to care (vs. has not received), confident to care (vs. not confident), confident in using PPE (vs. not confident), need training (vs. does not). Reference categories: female gender (vs. male), age (vs. over 50 years), Victoria (vs. other states), has prior mental health condition (vs. none), experienced family or friend infected with COVID-19 (vs. none), regional location (vs. metro), altered household income (vs. no change), has concerns about household income (vs. does not), number of years experience (vs. over 15), occupation (vs. medical), frontline area (vs. emergency department), currently working with COVID-19 (vs. negative response). * Other for the frontline area included people working in paramedicine, radiology, pharmacy, pathology and clinical laboratories, or other areas.

## Data Availability

Data available upon reasonable request to the corresponding author.

## Supplementary Materials

The following are available online at https://www.mdpi.com/article/10.3390/ijerph18179263/s1, Table S1: Predictors of mental health outcomes. Supplement S1: Future Proofing Frontline Healthcare Workers in Times of Pandemic and Other Crises.
