# Supplementary material for: Occupational Disruptions during the COVID-19 Pandemic and Their Association with Healthcare Workers’ Mental Health
Source: Int J Environ Res Public Health. 2021 Sep 2;18(17):9263. doi: 10.3390/ijerph18179263 (PMC8431156; doi:10.3390/ijerph18179263)
Supplement: Supplementary file 1 [file ijerph-18-09263-s001.zip › table S1.pdf]

International Journal of
Environmental Research and Public Health

MDPI

Supplementary Materials:

Supplementary Table 1: Predictors of mental health outcomes

Characteristics	Anxiety		Depression		PTSD		Depersonalisation		Emotional Exhaustion		Personal Achievement	
	OR (95% CI)	p	OR (95% CI)	p	OR (95% CI)	p	OR (95% CI)	p	OR (95% CI)	p	OR (95% CI)	p
**Professional Predictors**												
Increased Unpaid Hours		N/A	0.98 (0.81-1.19)	0.863		N/A		N/A	1.35 (1.11-1.64)	0.003	1.27 (1.05-1.53)	0.015
No Change		N/A	0.87 (0.74-1.03)	0.096	0.79 (0.70-0.90)	0.001	0.96 (0.85-1.09)	0.520	1.00 (0.86-1.17)	0.998	0.86 (0.74-1.01)	0.062
Redeployed		N/A	1.24 (1.03-1.50)	0.022	1.15 (0.97-1.36)	0.098	1.02 (0.87-1.19)	0.831		N/A		N/A
Role changed		N/A	1.17 (0.99-1.38)	0.065	1.42 (1.23-1.64)	<0.001		N/A	1.26 (1.08-1.47)	0.003		N/A
**Confident to Care for people with COVID-19**												
unconfident	0.98 (0.77-1.23)	0.838	0.99 (0.77-1.26)	0.902	1.02 (0.82-1.26)	0.893	1.15 (0.92-1.43)	0.225	1.26 (0.98-1.62)	0.074	1.11 (0.88-1.39)	0.402
confident	0.91 (0.75-1.11)	0.364	0.86 (0.70-1.05)	0.135	0.83 (0.70-0.98)	0.032	1.13 (0.94-1.36)	0.181	0.97 (0.80-1.18)	0.764	1.01 (0.84-1.23)	0.897
**Confident in Using PPE**												
unconfident	1.21 (0.90-1.62)	0.203	0.84 (0.62-1.16)	0.290	1.05 (0.79-1.39)	0.728	1.17 (0.88-1.56)	0.278	1.31 (0.95-1.81)	0.099	0.90 (0.67-1.20)	0.469

confident	0.79 (0.61-1.02)	0.068	0.89 (0.68-1.17)	0.404	0.84 (0.66-1.07)	0.148	1.05 (0.82-1.34)	0.701	1.20 (0.92-1.57)	0.187	1.31 (1.01-1.68)	0.039
Need Training	1.047 (0.907-1.208)	0.530	1.044 (0.900-1.213)	0.568		N/A	1.21 (1.06-1.38)	0.005		N/A		N/A
**Perception of workplace**												
Communication received	0.874 (0.713-1.071)	0.194	1.012 (0.815-1.255)	0.917	0.893 (0.736-1.084)	0.253	0.695 (0.573-0.843)	<0.001	0.902 (0.714-1.138)	0.384	1.29 (1.05-1.58)	0.015
Organisational support	0.46 (0.38-0.56)	0.001	0.52 (0.42-0.63)	0.001	0.59 (0.49-0.71)	0.001	0.62 (0.51-0.75)	<0.001	0.43 (0.34-0.55)	<0.001	1.38 (1.13-1.68)	0.001

PTSD: post-traumatic stress disorder.

Outcome comparators: anxiety, depression, PTSD, depersonalisation, emotional exhaustion (moderate to severe vs none to mild), personal achievement (moderate to high vs low).

Reference categories: increased unpaid hours (vs no change), no change (vs changed), redeployment (vs negative response), role change (vs no change), confidence to care (vs neutral), confidence in PPE (vs neutral), needs training (vs does not), perception of workplace (somewhat or very satisfied vs neutral or unsatisfied)
